# Social Relations, Community Engagement and Potentials: A Qualitative Study Exploring Resident Engagement in a Community-Based Health Promotion Intervention in a Deprived Social Housing Area

**DOI:** 10.3390/ijerph17072341

**Published:** 2020-03-30

**Authors:** Abirami Srivarathan, Rikke Lund, Ulla Christensen, Maria Kristiansen

**Affiliations:** 1 Faculty of Health and Medical Sciences, Department of Public Health, Section of Social Medicine, University of Copenhagen, Øster Farimagsgade 5, 1014 Copenhagen K, Denmark; rilu@sund.ku.dk (R.L.); ulch@sund.ku.dk (U.C.); 2 Faculty of Health and Medical Sciences, Center for Healthy Aging, University of Copenhagen, Blegdamsvej 3B, 2200 Copenhagen N, Denmark; makk@sund.ku.dk; 3 Faculty of Health and Medical Sciences, Department of Public Health, Section for Health Services Research, University of Copenhagen, Øster Farimagsgade 5, 1014 Copenhagen K, Denmark

**Keywords:** health promotion, interventions, community participation, deprived social housing areas

## Abstract

Emerging evidence points towards a lower quality of life, fragile social relations and suboptimal health behavior and status of residents living in social housing areas characterized by ethnic diversity and socioeconomic deprivation. Community-based health promotion interventions developed in collaboration with the target group and adjusted to the local context can affect the acceptance of and engagement in such interventions. However, few studies have investigated the potential of community-based interventions in deprived social housing areas. This study explores residents’ perspectives on engagement in a community-based health promotion intervention focusing on enhancing social relations. The study builds on qualitative methods including participant observations combined with pre- and post-intervention interviews with a selected group of residents (*n* = 9). Data were thematically analyzed with focuses on participation in an everyday life context, concepts of othering, and territorial stigmatization. Engagement in the intervention was motivated by the need to establish and enhance social relations, and to explore the world outside the housing area. However, barriers including cultural and language differences among residents, and competing contextual factors, challenged engagement. We conclude that participatory community-based interventions have a potential to enhance social relations in deprived social housing areas. However, adequate support and efforts to overcome the identified barriers are needed.

## 1. Introduction

In recent decades, there has been an increasing focus on the significance of residential areas in terms of the effect on health, wellbeing and social relations [1,2,3]. Residential areas with access to adequate housing, green spaces, formal and informal social and healthcare services, together with trusting and secure relationships with fellow residents, positively affect the health and wellbeing of residents through the course of life [1,2,4,5,6]. Social relations are essential to health and physical function, and successful social relations have a significant impact on both the physical and mental health and overall wellbeing of individuals [7,8]. Studies have demonstrated that the health and wellbeing of individuals vary according to local community characteristics, resulting in residents in deprived social housing areas having higher morbidity and mortality rates than residents in other, less deprived, areas [1,4,9,10]. The term social housing area used here refers to non-profit housing in Denmark intended for everyone, regardless of income and other social characteristics [11]. The housing comprises rental apartments and houses that are distributed based on a waiting list. Also, studies have shown the impact on health and wellbeing of the stigmatization caused by living in deprived social housing areas [12,13]. Additionally, residents from deprived social housing areas are less likely to engage in health promotion interventions compared to individuals of a higher socioeconomic position [9,14]. The observed differences in engagement in health promotion interventions are a result of a complex interplay of individual and contextual factors including distrust or fear of authorities, language barriers, and lack of information about and awareness of health promotion interventions [3,9,15,16,17,18]. Deprived population groups may be disempowered and unable to engage in traditional health promotion interventions, since these encourage individuals to exert control over their own health, often through a focus on advancing knowledge of risk factors [19,20]. Nevertheless, studies concerning recruitment approaches attempting to reach deprived population groups have found that working with residents of deprived social housing areas can enhance the acceptance of and engagement in health promotion interventions [3,9,15,21,22,23]. This entails that residents, in collaboration with formal and informal organizations in the deprived social housing areas, are involved in the design and implementation of the health promotion intervention [3,16,22,23,24]. However, regarding residents of deprived social housing areas specifically, little is known about the experiences of engagement in community-based health promotion interventions that are based on participatory approaches.

**Figure 1:**
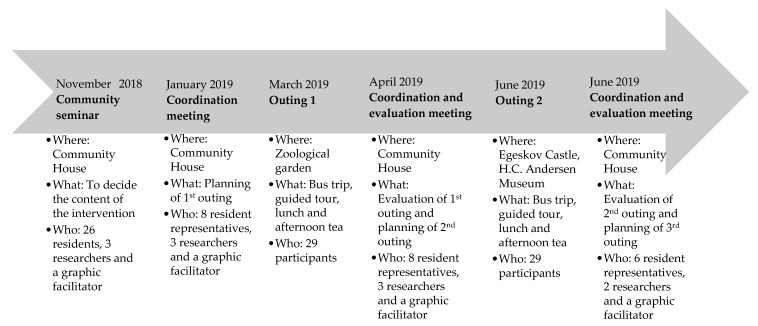
Co-creation process and intervention components.

The research project entitled ‘Health, wellbeing and social relations in a changing neighborhood’ examines the effects of large-scale structural changes on health, wellbeing and social relations in a deprived social housing area in Denmark from a longitudinal perspective. A more detailed description of the overall study has been published elsewhere [25]. By the application of multiple methods and different approaches to user engagement throughout the project period, the project focuses on empowerment and recognition of the residents in the housing area [25]. In the qualitative intervention study reported here, we explore residents’ perspectives on engagement in a community-based health promotion intervention focusing on the desired outcome of enhancing social relations, an intervention developed as part of the study. Engagement is defined here as ‘experiences with participation in the intervention and commitment to future interventions’. The community-based intervention was developed on the solid foundation of detailed knowledge from a previously collected ethnographic study, which, in turn, was based on qualitative interviews among the two largest resident groups (Danes and Turks), in combination with a baseline survey questionnaire from 2018 on health, wellbeing and social relations. The intervention was designed at a community seminar with residents, aimed at those above the age of 45 years, in a close collaboration between researchers and a graphic facilitator. Engagement was the key foundation for the development and implementation of the intervention. The use of graphic illustrative tools was included to enhance dialogue between researchers and residents [26]. After a process of co-creation, the residents who attended decided that the goal of the intervention was to enhance social relations and sense of belonging through health-promoting activities that were community-based, participatory and diversity-sensitive. To fulfill this aim, residents determined that the content of the health promotion intervention should consist of social outings to different sites and historical landmarks in Denmark. A working group of eight residents volunteered to participate in the planning of the intervention, in collaboration with three researchers and the graphic facilitator. Even though the destinations for the different outings varied, the structure of the planning stage of the intervention remained the same: namely that the eight resident representatives decided on the destination for each outing. Figure 1 illustrates a timeline of the co-creation process and the components of the intervention. 

### Aim of the Study

The aim of this qualitative sub-study is to explore residents’ perspectives on engagement in a community-based health promotion intervention to enhance social relations in a deprived social housing area. The primary research questions are:

1. What motives do residents have for engaging in the intervention, and what do they perceive to be the outcomes of their engagement?
2. What barriers and what potentials for improvement are identified in relation to resident engagement in the intervention?

## 2. Materials and Methods

### 2.1. Study Setting

This study was performed within a deprived social housing area in a suburb of Copenhagen, Denmark between February and July 2019. This social housing area was constructed in 1972 and comprises a total of 915 apartments, accommodating nearly 2600 inhabitants [25]. The built environment reflects the modernist mass housing trend of the post-war decades, characterized by a high density of dwellings and uniform high-rise buildings, all planned and constructed as a single entity [27]. Consequently, this housing area appears to be physically segregated from the surrounding local area and society. Furthermore, green spaces and outdoor facilities for residents to socialize in and utilize are sparse [25]. As in other deprived social housing areas in Denmark, socioeconomic vulnerability is profound in this area, with low levels of educational achievement, high unemployment and low average household income [25]. In addition, there is considerable ethnic diversity in the social housing area, with approximately 50 different nationalities represented. For the past three decades, a wide range of social and health care initiatives such as employment creation and locally anchored health promotion interventions have been implemented, supported by substantial funding in order to improve health, wellbeing and overall living conditions of residents. Furthermore, physical conditions have been improved by the renovation and modernization of the built environment. However, despite these various efforts, the deprived social housing area still faces challenges related to social and local environmental dynamics such as the proportion of children receiving lower grades in primary schools, the proportion of adults with no formal education, and the low income levels, along with the absence of green spaces and the poor quality of the housing [28]. A national policy directive from December 2018 required this particular deprived social housing area to undergo large-scale structural changes: firstly to the built environment; and secondly to the composition of the resident group with regard to country of origin and socioeconomic status. This will result in the demolition of a certain number of apartments before 2030 [25].

### 2.2. Selection of Participants

The inclusion criteria for participation in the present study were based on: (1) age (45 and above was the target group for participants in the intervention), (2) country of birth (including only residents born in Denmark and Turkey as these nationalities represent the largest groups in the deprived social housing area, and so as to be able to balance the number of languages represented with the resources available for the study), (3) place of residence (since the intervention was limited to only residents in that particular deprived social housing area), and (4) participation in the intervention in order to comply with the aim of the study. As part of an effort to ensure variation among the participants of Danish and Turkish origin enrolled in the study, we applied different recruitment strategies. Participants were recruited during a number of different formal and informal events in the deprived social housing area. This included recurring breakfast cafés and needlework classes organized by the residents’ association, as well as individual encounters with potential participants. Snowball sampling was also used in order to get in contact with more vulnerable residents in the different target groups. During recruitment, it was important that close collaboration was maintained with key community figures such as members of the resident representative committee, chairpersons of local associations and other volunteers. The importance of this close collaboration emerged, for example, when the first author was advised to recruit female participants of Turkish origin in the needlework association, since a large number of these female residents supported the association. Field notes focusing on resident interaction, including dynamics and challenges, were taken throughout the recruitment period as these provided additional insights into the different social groupings and gatherings in the housing area. Recruitment of participants was continuously ongoing up to the occurrence of the intervention during the study period until enough information power in the data material was achieved [29]. The final sample of participants consisted of nine residents born in Denmark (7) and Turkey (2) with Danish and Turkish as the majority languages (Table 1). A large proportion of the participants were either retired or received welfare handouts and suffered from comorbidities including chronic diseases such as hypertension, elevated cholesterol levels, non-insulin-dependent diabetes and osteoarthritis. The average time of years lived in the housing area among the residents included in this study was 22 years.

### 2.3. Data Collection

Data were collected through 18 individual semi-structured interviews over a time period of six weeks with nine pre-intervention interviews and nine post-intervention interviews focusing on resident engagement in the two social outings described in Figure 1. Pre-intervention interviews were carried out two weeks prior to the intervention, and post-intervention interviews were conducted a month after participation in the intervention in order to assess the impact of engagement in the intervention on social relations. The pre-intervention interviews were conducted in order to elucidate needs and motives for engagement among residents in an everyday life context. The post-intervention interviews focused on perceived outcomes of engagement, identified barriers and perspectives on future engagement in similar interventions. The themes included in pre- and post-intervention interviews and observation guides are shown in Table 2. Themes such as ‘health and wellbeing’, ‘the housing area’, ‘social relations’ and ‘the intervention’ in the pre-intervention interview guide served the purpose of uncovering the needs and motives for engagement, whereas themes such as ‘social interactions’, ‘evaluation of the intervention’ and ‘perspectives on future interventions’ in the pre-intervention interview guide were included to illustrate the perceived outcomes and barriers of engagement. Interviews lasted between 40 and 97 min with the average duration of interviews being approximately 65 min. Interviews were performed in a setting based on the participant’s own choice, with 10 of the interviews conducted in the homes of the participants and eight in facilities belonging to local community associations. Observations related to the intervention occurred over a period of six months within and outside the housing area. Furthermore, we observed encounters and interactions among residents that were later included in interviews, and our observations of residents who were not interviewed were documented through field notes.

### 2.4. Data Analysis Procedures

All interviews were audio recorded and transcribed verbatim and field notes were written out. The data material consists of 460 standard pages of transcriptions and 47 standard pages of field notes. A thematic approach applying a strategy of systematic text condensation was used to analyze the data [30]. Interviews and field notes were read through in order to become familiar with the data, develop an impression of the text as a whole and identify themes. The data were then coded into meaningful units and summarized into main themes exploring the motives and experiences of residents engaging in the community-based health promotion intervention. Field notes were also used to contextualize findings. During the coding of the data material, the first and last author constructed thematic networks, which were subsequently compared to obtain a broader view of the data material as well as to ensure a wider understanding of our findings. After reviewing the themes and redefining them, subthemes related to each main theme were discussed. Throughout the analysis, an exploratory approach was applied, with the ambition of presenting selected patterns that elucidate the study aim, rather than to cover the whole range of the phenomena [29]. Regarding the establishment of social relations, the concept of othering was used as an analytical starting point to tease out the cultural processes of identity formation among residents of different countries of origin [31]. Territorial stigmatization presented by Loïc Wacquant inspired the analysis of ways in which political decisions and actions causing lack of stability and structural changes influence engagement in health promotion interventions among residents living in areas of deprivation [32,33].

### 2.5. Ethical Considerations

The study was recorded in the University of Copenhagen’s joint record of bio banks and research projects containing personal data (journal number: 514-0183/18-3000) and conducted in compliance with the ethical principles for medical research presented in the Helsinki Declaration [34] and in accordance with The General Data Protection Regulation. Data material comprising audio files, transcriptions and field notes, were securely stored and managed. The participants were informed in their native language, both orally and in writing, about the objectives of the study, research ethics, and the right to withdraw at any time during the study period. A professional interpreter was offered to all participants with Turkish background, and was subsequently involved in four interviews. Participants were encouraged to contact the interviewer after the interviews to express any doubts and seek clarification. In a number of situations, the interviewer helped participants navigate relevant social and health care services as participants expressed unmet needs.

## 3. Results

**Figure 2:**
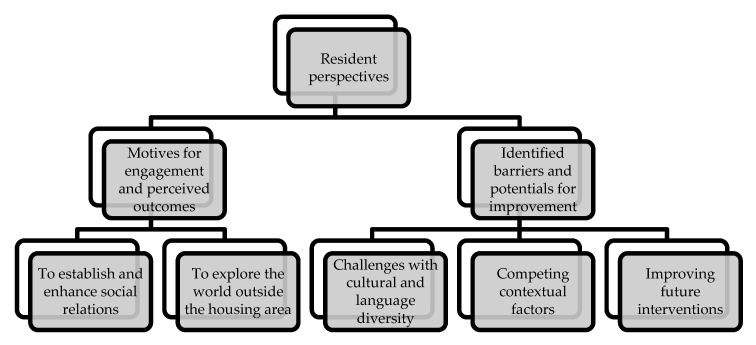
Structure of thematic network.

Overall, five themes emerged from the analysis of the transcripts and field notes. These five themes were grouped into two broad categories, (1) motives for engagement and perceived outcomes and (2) identified barriers and potentials for improvement, with both categories shaping resident perspectives on engagement in the community-based health promotion intervention. Figure 2 illustrates the structure of the thematic network and the identified themes. Each of the five themes is explored below with the use of illustrative quotes and field notes to reflect the perspectives of the participants.

### 3.1. Motives for Engagement and Perceived Outcomes

#### 3.1.1. To Establish and Enhance Social Relations

The participants expressed a need to establish new, and enhance existing, social relations with other residents in the housing area as important in terms of motives for engagement in the intervention. The vast majority of participants were either retired or received social welfare benefits and thus spent most of their everyday life in their homes. A few participants were married, but their spouses were affiliated to the labor market and therefore they were often left alone during daytime hours. All participants had adult children who had left home years ago and started their own families. Gizem described her everyday routines and emphasized the extra amount of time available as a result of her life circumstances:

*“The children are grown-ups now, and they have left home. My husband goes to work in the morning, and comes back in the evening. I am home alone during the daytime.” *

*(Gizem)*

The participants of Danish origin described their social network as consisting of close family members and a few friends mostly living outside the housing area, whereas participants with Turkish origin reported having a larger network within the housing area. Observations also highlighted these differences, as female residents of Turkish origin were involved, along with other residents of Turkish origin, in the various local associations within the housing area. A wish to establish new relations and connect with other residents by engagement in the intervention was especially pronounced for the female participants of Danish origin:

*“So I genuinely hope that I get to know new people during this outing to the zoo. I have to admit that this is the purpose of my engagement in the intervention.”*

*(Karen)*

In addition to the perspective presented by Karen, some participants expected that engagement in the intervention would result in interactions with residents of different origins than their own. Rose said:

*“If we get some of the Turkish women on board, I think it will be good for us. I believe that we need to include them a little more. Also, the other migrants in this housing area.”*

*(Rose)*

During both intervention observations and pre-intervention interviews, it appeared that the establishment of social relations was a key motive across the various different resident groups. Apart from establishing new relations, residents also perceived their engagement in the intervention as a possibility to enhance and strengthen existing relations. Some residents knew each other in advance based on previous encounters of a more sporadic nature. In one participant’s words:

*“You see, Karen is also over 45, so I thought that it could be very nice if she wanted to come along. Then we could talk to each other, and get to know each other better during the outing. So, I think it is interesting that in that way people can get to know each other better.”*

*(Vagn)*

For Vagn, Rose and Karen, engagement in the intervention was expected to be a platform for both increasing the quantity of social relations by expanding their social network, and also enhancing the quality of existing social relations in the housing area. Through post-intervention interviews, other participants of Danish origin shared this perspective related to expanding their social network, as they described, after their engagement in the intervention, that they now had encounters with other residents in different places in the housing area:

*“I met one of the women from the housing area in a supermarket the other day, and she said, ‘Hello’. What is more, she opened her arms. We talked about the outing, and our opinions about it, and we agreed that it was a good outing.”*

*(Kaj)*

Kaj clearly experienced the encounter with the other resident as socially positive, and an occasion to evaluate the intervention as also being positive. This perceived outcome was also observed in the breakfast café following the intervention, where participants greeted each other warmly, and chatted about the benefits they felt they had gained from having participated in the outing. Participants also discussed the potential of these interventions to further gather and unite all residents. As described by one participant:

*“I think that these outings can be a stepping-stone to make progress. But as I told you, I would probably like it if a club was set up for everyone here in this housing area.”*

*(Lone)*

After engagement in the intervention, Lone also believed that it could serve as a “stepping-stone” for further and future coming together of residents in the housing area, added to her idea of creating an inclusive association for all residents.

#### 3.1.2. To Explore the World Outside the Housing Area

Even though residents of Danish origin reported having social relations outside the housing area, all the residents, irrespective of country of origin, spent a large part of their everyday life inside the housing area, seldom experiencing the world outside the housing area, especially during working days. Residents described how engagement in the intervention was a way of breaking out of humdrum everyday life:

*“It is about getting out and seeing something new, instead of sitting here and staring at these four walls. And to get to experience something.”*

*(Wilma)*

This need to explore the world outside the housing area was particularly noticeable among female residents regardless of their country of origin, and of whom none in our study were attached to the labor market. Two out of three male participants, Kaj and Martin, were still attached to the labor market, and they described their everyday life as highly structured around their working hours. An average working week of 50 h caused a limited window of opportunity to get outside of the housing area, particularly during weekdays, when the evenings were spent there. In one participant’s words:

*“You need something to happen once in a while. You need to get a bit outside the corner down there [laughs] and experience that: ‘God, there is also a world outside this place!’ And then there is the thing about getting a break in your weekly routine. Walking around in the housing area is all very well, but you cannot avoid that it somehow affects you that every time you stare out of the window you look [whistles] directly down at the sidewalk there”.*

*(Kaj)*

Both Kaj and Martin perceived their engagement in the intervention as a break from everyday life. For all residents, engagement in the intervention was described as a much-needed initiative to experience something new in a monotonous everyday life characterized by routines. Even though the residents described their everyday life as ordinary and mundane, these illustrations of the residents’ own descriptions of everyday life are important, since these emphasize what residents value as essential to their lives. During observations in the housing area prior to the intervention, residents’ eagerness and curiosity to acquire new knowledge and experiences were noticeable. Throughout the observations in the community house café and the needlework association, residents often expressed looking forward to expanding their horizons by engaging in the intervention. Their eagerness and curiosity were also verbalized during pre-intervention interviews:

*“I am very excited. I love it. And, as old as I am, just as curious to try something new. I am filled with zest for life. In Turkish, we have a saying that sounds something like this: ‘It is not the people who read a lot that also know a lot, but it is the people who travel widely who know the most. You have to get out and experience new places, and process the experiences in your mind [laughs]. We need some activities that bring us out [of the housing area] and provide us with new knowledge”.*

*(Dilara)*

A distinction between residents of Danish origin and residents of Turkish origin emerged in relation to the theme of exploring the world outside the housing area. The pre-intervention interviews with Dilara and Gizem, as well as the observations made in the needlework association, showed that for most residents of Turkish origin, engagement in such outings was a first-time experience, since they spent most of their everyday life inside the housing area. In the cases of Dilara and Gizem, they were granted residence in Denmark through family reunification. Both women described that they had had little experience in the labor market and other societal institutions, and were overall less included in the various associations and organizations in the surrounding area. Conversely, all residents of Danish origin had some experience with engagement in such interventions, and therefore expected engagement in the intervention would refresh their memory:

*“So I am looking forward to getting to revisit Egeskov Castle. Because it is probably 10 to 15 years ago, since I was last there. I get the usual experience of experiencing some culture. I am also a fan of historic stuff, and I will of course enjoy experiencing that again, because I have not seen it for some time now.”*

*(Martin)*

For residents such as Martin, Kaj, Rose and Wilma, engagement in the interventions was perceived as an opportunity to revisit different locations and furthermore to experience them from a new point of view. This perspective was repeated during the-post intervention interviews with these residents, where they described that they experienced the intervention as a “trip down memory lane” to locations that they had visited decades ago in their childhood. For residents such as Dilara and Gizem, engagement in the intervention was perceived as a glimpse into new learning about the world outside the housing area and an expansion of their horizons.

### 3.2. Identified Barriers and Potentials for Improvement

#### 3.2.1. Challenges with Cultural and Language Diversity

In general, participants expressed satisfaction and joy regarding their engagement in the intervention during post-intervention interviews and observations, and emphasized the importance of arranging such interventions for residents of the housing area. However, as the interviews unfolded, it appeared that most participants experienced challenges in relation to their attempts to reach out to residents of different ethnic origin from their own, and who had another native language. This challenge was also observed during the intervention itself, where residents often tended to gather with others from their own country of origin. Gizem, born in Turkey, described how she felt rejected when she tried to reach out to a female resident of Danish origin, while she was waiting to get onto the bus before departure for the zoo:

*“The Danes did not look at us at all. I asked a woman whether she was also going on the outing, and she did not even look at me. It is as if they are afraid of us. I get this feeling. The more you try to get closer to the Danes, the more you get pushed away.”*

*(Gizem)*

The episode was also confirmed by participant observation. This feeling of rejection caused doubt among the residents as to whether interventions such as these could contribute at all to the establishment of social relations among residents of different origin. In trying to create new relations in their everyday life in the housing area, the participants routinely experienced barriers related to cultural and language differences, and particularly during their engagement in the intervention. In one participant’s words:

*“I am very doubtful about [being able to] make friends with residents of different origin. Because there was a core group of especially Turkish women who stayed by themselves. I think the ethnic Danes are more open to talking with other people. I am at least open to talking with other people. But it seems as if they [Turkish women] are a more closed circle.”*

*(Vagn)*

Following the intervention, Vagn expressed skepticism towards establishing interventions that could bring residents of different origin together based on his current experiences in relation to approaching residents of Turkish origin, especially females, as these female residents was perceived to be a more closed circle. This gender aspect was not only shared by the other male participants of Danish origin in the study, but also by two female participants of Danish origin, who had noticed that female residents of Turkish origin never spoke to male residents of Danish origin. A common feature of the perspectives presented by Gizem and Vagn is that they both described how residents different than themselves were dismissive of interaction and communication. The need to dissociate themselves from the represented type of residents by ascribing them problematic and negative behaviors can be seen as an act of othering. Both Gizem and Vagn created or underlined their own respective identities through their attempts to reach out to residents they did not know (to those from different countries), and then to distance themselves from the negative and reserved behavior displayed by those residents of different origin than themselves.

#### 3.2.2. Competing Contextual Factors

During the study period, a decision was made to demolish a specific number of apartments in the housing area to comply with a recent law made by the government [35,36]. Political decisions such as this reflect territorial stigmatization, which had a negative effect on the engagement in future interventions among all residents, regardless of their country of origin. During the pre-intervention interview, Karen declared that she was highly motivated to engage in future interventions in order to meet other residents and establish new social relations in the housing area. However, during the post-intervention interview, Karen explained that her level of engagement had declined over the past six weeks between the pre-intervention and post-intervention interview due to political decisions regarding structural changes in the housing areas:

*“I risk losing my apartment, because it might be demolished. I cannot stop thinking that I need to find a new place. I also think: why I should get involved [in this housing area]? I mean, it does not matter [if I need to move] anyway.”*

*(Karen)*

Besides the uncertainty and insecurity caused by the structural changes in the built environment, residents described some of the psychosocial aspects of their everyday life in the housing area as factors acting as barriers to engagement. For some participants, especially the female residents of Turkish origin, other challenges in the housing area, such as unemployment and crime among young residents, took up much headspace. In light of these challenges, interventions on health, wellbeing and social relations among middle-aged and older adults were not given the highest priority. As described by one of the participants:

*“The children and young people do not have any education in our housing area. They are involved in some sort of criminality. They [the public sector] are not protecting our young people. What do they want to do with us [older adults]? They need to take care of the young people”.*

*(Gizem)*

Although residents expressed a general willingness regarding engagement in the intervention, other factors such as societal legislation, structural changes and contextual challenges related to the reality of the everyday life in the housing area thus affected their level of engagement.

#### 3.2.3. Improving Future Interventions

During the post intervention interviews, participants reflected on strategies to overcome some of the barriers presented above. In general, participants irrespective of their country of origin emphasized a need to improve possibilities for interaction among residents of different origin in future interventions. Language barriers were highlighted as being of particular concern: participants described that these should be addressed in order to enable their engagement with all residents. Some participants considered alternative approaches to the intervention in order to comply with their wish to establish social relations with residents of different origin:

*“We need some activities where you are able to team up with other residents, so I would be together with some female residents of Turkish origin. Then we would have to talk together. Then we would be forced to spend time together [with the others].”*

*(Vagn)*

Vagn recommended that future interventions should, to some extent, force residents to communicate with each other, and should additionally create “teams” of residents from different countries during the intervention, to facilitate interaction between all residents. This recommendation may be seen as an attempt to challenge the process of othering among residents. During post-intervention interviews and informal conversations during observations, residents expressed frustration and desperation towards governmental institutions specifically, and wider Danish society in general. Ever since Gizem arrived in Denmark more than 30 years ago, her place of residence had been in the housing area. Over the past decade, she had experienced that despite the many efforts to address challenges related to socioeconomic vulnerability in the housing area, the rhetoric continued to be negative:

*“For the past 10 years there have been many, many interventions. However, they have always found an excuse for calling the housing area a ‘ghetto’ or giving it a bad reputation, but the housing area is not at all like that.”*

*(Gizem)*

Residents such as Gizem experienced that the housing area and their everyday life in it, were associated with stigma, which in addition created a feeling of exclusion from the rest of society surrounding the housing area. Furthermore, Gizem described how the public sector had already implemented several out of the ordinary initiatives justified by the socioeconomic vulnerability in the housing area. These experiences were shared by other residents, who described that interventions such as these resulted in the destabilization and marginalization of residents, causing territorial stigmatization. Based on these perspectives, future interventions should focus on dispelling territorial stigmatization created by the wider society towards the housing area. By opening the housing area up to the surrounding community and creating encounters between general citizens and residents, it is possible that the stigma associated with the housing area and its residents can be challenged. Furthermore, interventions addressing territorial stigmatization could be rolled out more widely to contribute to the inclusion of not only this particular housing area, but also similar areas of society in Denmark and internationally.

## 4. Discussion

In this study, we have explored resident perspectives on engagement in a community-based health promotion intervention, building on participatory approaches aimed at enhancing social relations among residents of a housing area with manifest deprivation. We identified that engagement in the intervention was driven by motives such as to establish and enhance social relations with fellow residents, and a need to explore the world outside the housing area. However, engagement in the intervention was challenged by barriers related to cultural and language diversity among residents, together with competing contextual factors (demolition plans, and territorial stigmatization), resulting in doubtful engagement in future interventions. These findings correspond with other studies [9,15] which emphasize a need for context-specific and participatory approaches to ensure that engagement in community-based health promotion interventions in areas of deprivation can be maintained.

### 4.1. Social Relations and Community Engagement as Motives and Perceived Outcomes

Challenges related to the acceptance of and engagement in health promotion interventions among residents in deprived areas of social housing have previously been identified [9,15]. Nevertheless, our study shows a general positive acceptance of and engagement in community-based interventions among residents living in a deprived area of social housing. Engagement in the intervention was driven by a desire to establish and enhance social relations. Our findings, in accordance with other studies, demonstrate that the social relation aspect is an important motive for engagement in community-based health promotion interventions [21,37,38]. In investigating the motivation for residents of deprived areas of social housing to engage in health promotion interventions, a systematic review of strategies to improve engagement of hard-to-reach older adults emphasized that participants preferred group-based health promotion interventions compared to individual sessions because of their social element [21]. The establishment of new relations to fellow residents and the enhancement of existing relations among residents in our study are health promoting, since positive social relations are favorable for both the physical and mental health and overall wellbeing of individuals [7,8]. In terms of perceived outcomes of engagement in health promotion interventions, a rapid review of experiences of community engagement showed that participants described the establishment of social relationships with fellow participants as a positive outcome of their engagement in health promotion interventions in local communities [37]. In another Danish ethnographic study, the residents interviewed felt that their engagement in activities in the housing area was an investment in improving the social relations of their everyday life [38]. As our own findings indicate, social cohesion and further desire to invest in local association activities in the housing area are, as reported by residents, additional outcomes of engagement in community-based health promotion interventions [39].

### 4.2. Cultural and Language Diversity as Barriers to Meeting other Residents

Even though the participants in our study expressed the desire to meet with and to establish relations with residents from different countries of origin, they identified challenges related to cultural and language diversity. Consequently, this diversity among residents led to suboptimal encounters or at worst no meetings at all between residents of different ethnic origin. Our study, in line with other studies, found that participants in health promotion interventions are inclined to interact only with other participants who shared the same characteristics as themselves, such as country of origin [40]. This apparently natural inclination to meet with people of a similar disposition can challenge future engagement in, and thereby the sustainability of, community-based health promotion interventions [13,38,40]. Furthermore, these various studies have found that ethnic stereotyping and othering processes were present among all the different resident groups in the social housing areas. Participants often distanced themselves from other participants of different country of origin by ascribing these negative behaviors to resident groups different than themselves. Additionally, participants tended to praise their own behavior and that of residents similar to themselves, while exaggerating differences between the behavior of themselves and of other residents who were dissimilar to themselves, thereby ascribing negative behavior patterns to these others. [13,38,40].

Our findings correspond well to the abovementioned studies in that, in our study, groups of residents with both Danish and Turkish origin perceived suboptimal encounters or none between residents of a different country of origin. These perceptions were based on dismissive behavior exhibited by the counterpart, intensified by language barriers. In addition to challenges related to cultural and language diversity, cultural beliefs and preferences regarding gender were also stressed as being obstacles to encounters between the residents in our study. During post-intervention interviews, the male participants of Danish origin in particular described that the establishment of relations with female residents of Turkish origin was a particular challenge. These male participants experienced that the female residents of Turkish origin preferred to meet only with other female residents of same origin. This finding is supported by a systematic review that identified cultural beliefs and preferences regarding mixed-sex encounters as a barrier to engagement in health promotion interventions [21].

### 4.3. Sustainability of the Intervention and Recommendations for Future Interventions

Our findings point towards a number of contextual circumstances that should be considered in relation to residential engagement in health promotion interventions in deprived areas of social housing. Lack of social stability caused by the structural and structural changes in the housing area was reported to constrain interaction among residents and also their ability to engage in civic activities, or to participate in recreational activities [41]. Furthermore, other community issues such as crime rates affected the residents, which caused disengagement in local community activities among residents [42]. As described in our findings, residents in deprived areas of social housing are often exposed to numerous health promotion and social care interventions as part of strategies to improve the quality of life among the residents [38]. Participation fatigue has been identified as a barrier for future engagement in the local community, since the interventions were perceived as a strategy to rescue the housing area and “save” the residents [38]. This type of framing of health promotion interventions could unintentionally stigmatize the housing area and the residents as a problem that needs special attention and interventions out of the ordinary [32,33]. The impact of territorial stigmatization on residential community engagement in deprived areas of social housing in a Danish context is equivocal. In accordance with our findings, it has previously been shown that territorial stigmatization affects social solidarity in the housing area and hinders future engagement in the local community [28,43]. Also, an ethnographic study found that residents in a deprived area of social housing are affected by territorial stigmatization in their everyday life to the point that they become sad, frustrated and angry [44]. However, this territorial stigmatization seemed *not* to affect the level of community engagement in local associations and civic activities among the residents interviewed in the housing area in this same study [44]. Our own study indicates a need for approaches based on the contextual circumstances found in this study in the future recruitment and delivery of health promotion interventions in order to ensure sustainability of these interventions in the housing area we studied. A greater involvement of existing infrastructure through historically collaborative partnerships or an influential community partner, such as locally driven associations by residents or the board of residents, holds a potential in terms of securing post-intervention sustainably of health promotion interventions [22,24]. An influential community partner, as previously mentioned, has many years of experience with designing and implementing community-based interventions in the deprived social housing area, and holds valuable insight about the needs and preferences of residents that can be an advantage for the development and sustainability of community-based health promotion interventions. The benefits of such extensive collaboration through community engagement are reported to be empowerment and improvement of social networking and self-efficacy skills among residents [24]. The territorial stigmatization identified in our study suggested that greater involvement of an influential community partner could be beneficial in terms of ensuring participant approval and support in future health promotion interventions [22,24].

### 4.4. Methodological Considerations

The findings reported in this study are derived from fieldwork that demanded a high level of local presence and trust-building in a deprived social housing area, and hereby the first author gained access to a hard-to-reach population group. This fieldwork consisted of a rich empirical dataset with many perspectives on motives for, and perceived outcomes on, engagement in a community-based health promotion intervention in an understudied population group. Our findings are situated in a particular time and context, and it is not our intention to generalize the findings to other groups and contexts. Nevertheless, we expect that some of the same motives and challenges regarding engagement in community-based health promotion interventions among residents living in similarly deprived social housing areas can be found. The present study is explorative, and further research in the field of engagement in community-based health promotion interventions to enhance social relations among residents living in deprived social housing areas should be initiated.

The concept of information power examines the amount of information that the data material contains and its relevance for the study [29]. Since the aim of the study is to explore resident perspectives on engagement in a community-based health promotion intervention, participants enrolled in the study had characteristics highly specific to the proposed aim, while at the same time presenting some variations in the experiences explored, such as sex, age and country of birth [29]. Factors such as sex, age, country of birth, employment status, marital status and duration of residence in the deprived social housing area differed among participants and naturally, this resulted in diverse life stories, different motives and barriers and varying experiences of engagement in the community-based health promotion intervention. Despite the differences among the study participants, some common characteristics were found. All participants suffered from one to several chronic diseases, and for some participants this had led to withdrawal from the labor market. Furthermore, all participants spent their everyday life in a deprived social housing area, which had been subjected to negative publicity by politicians and the media, resulting in territorial stigmatization.

The recruitment strategy comprising the use of gatekeepers had its advantages and drawbacks [45]. In some situations, the use of gatekeepers enabled a more trusting relationship between the researcher and the participant, while in other situations, personal tensions between the gatekeeper and the participant affected the initial establishment of contact with the participant. Furthermore, the recruitment strategy focusing on association activities in the deprived social housing area and snowball sampling resulted in an unequal study sample with a preponderance of participants of Danish origin. Since the findings indicate different perspectives among residents of Danish and Turkish origin, it is recommended that future studies focus to a greater extent on ethnic differences regarding engagement in community-based health promotion interventions in deprived social housing areas. As documented in other studies, there are several challenges related to the recruitment of ethnic minority population groups in health research [3,45]. During the recruitment of participants of Turkish origin, the first author experienced challenges including mistrust of authorities and fears over the use of personal information. The use of an interpreter during the interviews with residents of Turkish origin may have challenged the interaction between the researcher and the participant, since the natural flow of conversation was affected by and conditional on the translation process [46]. However, prior to the interviews the interpreter was informed about the study aim, research ethics and guidelines for interpretation, and the interpreter facilitated, in general, a trusting and nuanced interaction between the researcher and the participant.

### 4.5. Implications for Policy, Practice and Research

There is a need for a continued policy focus on enhanced and innovative approaches regarding health promotion interventions targeting residents living in deprived social housing areas. The findings of this study indicate the need for interventions addressing social cohesion and engagement across residents of different country of origin and native languages in order to ensure relevant health promotion interventions, and to “build bridges” between deprived social housing areas and the surrounding communities. Policies should acknowledge that community-based health promotion interventions in deprived social housing areas might be highly resource-demanding, as the establishment of relations between residents with different cultural and language background is time-consuming.

In relation to practice development, there is a need to develop interventions that force residents to interact with each other, so that residents might be challenged in their own perceptions of residents different from themselves, and thereby be able to gain a fresh impression of other residents. Through this new impression, residents may be enabled to overcome their perceptions of problematic and negative behaviors which they ascribe to residents with different country of origin compared to themselves.

This study has a longitudinal qualitative approach on the engagement in a community-based health promotion intervention among residents living in a deprived social housing area. However, a longer study period with follow-up interviews would be desirable in relation to exploring the long-term effects of engagement in the studied intervention. Furthermore, quantitative methods such as survey questionnaires would be useful to describe the need for, and current practices of, health promotion interventions on a larger scale across deprived social housing areas. This will enable the further development of community-based health promotion interventions and ensure that these interventions are relevant for residents living in these deprived social housing areas.

## 5. Conclusions

This study has explored perspectives on engagement in a community-based health promotion intervention to enhance social relations among residents living in a deprived social housing area. The findings indicate that the intervention holds a potential to enhance social relations among residents, since the primary motive for engagement was reported to be the social relations aspect. In line with this, the study points towards a further engagement in health promotion interventions in the housing area as a perceived outcome of engagement in the intervention. Furthermore, the residents’ needs to explore the world outside the housing area, and to take a break from everyday life characterized by routines, were identified as motives for engagement. However, a number of barriers related to cultural and language diversity, and challenges regarding contextual factors, have been pointed out, potentially affecting engagement in future health promotion interventions. Therefore, adequate support and efforts to overcome the identified barriers are needed to ensure feasible and relevant health promotion interventions in similar settings in order to encourage resident engagement.

## Figures and Tables

**Table 1:** Participant characteristics.

Participant Pseudonym	Age Categories in Years	Sex	Country of Birth	Employment Status	Marital Status	Duration of Residence in the Housing Area in Years
Gizem	50–59	Female	Turkey	Unemployed	Married	30–39
Lone	60–69	Female	Denmark	Unemployed	Single	10–19
Karen	50–59	Female	Denmark	Unemployed	Single	10–19
Vagn	70–79	Male	Denmark	Retired	Widowed	30–39
Dilara	60–69	Female	Turkey	Retired	Married	30–39
Wilma	60-69	Female	Denmark	Retired	Single	10–19
Rose	80–89	Female	Denmark	Retired	Single	20–29
Kaj	70–79	Male	Denmark	Employed	Single	<10
Martin	60–69	Male	Denmark	Employed	Single	10–19

**Table 2:** Themes included in observation and interview guides.

Themes Explored in Participant Observations	Themes Explored in Pre-Intervention Interviews	Themes Explored in Post-Intervention Interviews
**Non-human actors and context** Time available for interaction Space/location of the interventions **Human actors** Which groupings, divisions and positions take place? Atmosphere (formal/informal) Interactions between residents/researchers/other people **Communication** What is articulated before, during and after the interventions and what is left unspoken (everyday life context, need for activities, social relations)? Who brings up topics during the conversations and how are they responded to? **Body language** Silences and nonverbal reactions	**Background information** Age, marital status, significant others/children, level of education, employment **Health and wellbeing** Self-reported health Influence of illness on everyday life (physical, psychological, social consequences) **The housing area** Everyday life in the housing area Knowledge, usage and satisfaction of formal and informal health promotion activities **Social relations** Solidarity amongst residents Common identity of residents Perceptions of appreciation and tolerance of diversity in the housing area **The intervention** Immediate reactions of and perspectives on the intervention Pros and cons in relation to engagement Motives for engagement Expected outcomes of engagement	**The specific intervention** Memories of the intervention from beginning to end **Social interactions** Experiences with verbal and nonverbal interactions Experiences with language and cultural differences among residents Establishment of new relations Experiences with continued interactions after intervention **Evaluation of intervention** Satisfaction with amount of information received prior to the intervention Optimal/suboptimal conditions Improvements Perceived outcomes of engagement Accordance between expectations and perceived outcomes **Perspectives on future interventions** Engagement in future interventions Engagement in other activities in the housing area Association between motives, perceived outcomes and future engagement
